# Minimizing Guidewire Unwilling Passage and Related Perforation During Transradial Procedures: Prevention Is Better Than Cure

**DOI:** 10.3389/fcvm.2022.730648

**Published:** 2022-03-01

**Authors:** Lili Xu, Jiatian Cao, Meng Zhang, Hongbo Yang, Zheyong Huang, Yanan Song, Chenguang Li, Yuxiang Dai, Kang Yao, Xiangfei Wang, Feng Zhang, Juying Qian, Junbo Ge

**Affiliations:** ^1^Department of Cardiology, Shanghai Institute of Cardiovascular Diseases, Zhongshan Hospital, Fudan University, Shanghai, China; ^2^National Clinical Research Center for Interventional Medicine, Shanghai, China; ^3^Department of Cardiology, Xiamen Branch, Zhongshan Hospital, Fudan University, Xiamen, China

**Keywords:** coronary angiography, transradial procedure, knuckle guidewire, safety, efficiency

## Abstract

**Background:**

Current guidewires for transradial coronary angiography had defects of passage difficulty or branch injury. This study sought to investigate the safety and efficiency of a novel method of active knuckle-angle 0.035-inch hydrophilic guidewire in transradial coronary angiography.

**Methods:**

Patients undergoing a transradial coronary procedure in our team from August 2015 to June 2020 were retrospectively investigated. We compared the demographic and interventional characteristics of 1,457 patients receiving advancement of unmodified guidewires (Traditional group) and 1,322 patients receiving advancement of the knuckle guidewire (Knuckle group). Afterwards we included 239 patients and randomized them according to a random number table to either the unmodified or the knuckle guidewire to further confirm the efficiency and safety of knuckle guidewire advancement.

**Results:**

In the retrospective analysis, unwilling passage of guidewire into branches occurred more in the Traditional group than in the Knuckle group (9.5 vs. 0.08%, *p* < 0.001). Two patients in the Traditional group experienced guidewire-associated perforation. One patient was treated with covered stent for internal mammarian artery perforation, while the other was managed with compression for brachial branch perforation. In the randomized controlled study, unwilling passage of guidewire also occurred more in the Traditional group (10.8 vs. 1%, *p* < 0.001). Median duration of guidewire advancement from the sheath to aortic root significantly decreased from 33 seconds in the Traditional group to 21 seconds in the Knuckle group.

**Conclusion:**

Active knuckle angle guidewire represented a novel method to prevent unwilling passage and associated perforation with efficiency improvement and a reduction in radiation exposure.

## Introduction

Radial artery access has been widely applied as a default route for diagnostic and interventional coronary procedures with reduced vascular complications, shorter hospital stay and improved outcomes compared to the femoral approach (1–5). Effective hemostasis of the radial artery is due to its superficial course and small luminal caliber. However, anatomic variations of the radial artery and the S-shaped configuration of the subclavian-innominate-aorta axis often blocks J-tip 0.035-inch guidewire passage (6, 7). Some doctors prefer the J-tip guidewire which was originally designed for a large lumen in the transfemoral approach. It has some limitations in transradial access, such as the potential risk of perforation before the guidewire enters a large vessel and the safe J tip forms.

Another choice is an angle-tip 0.035-inch hydrophilic guidewire (Radifocus, Terumo, Japan) commonly used for its superiority in tortuosity and direction changes (8). However, it can glide into small side branches without appreciable resistance, increasing the risk of subintimal dissection and perforation (9, 10). Sometimes a side branch mimics the main artery due to its lengthy and parallel course (7, 11).

Therefore, difficulties in the passage of guidewires cannot be completely avoided and may cause different complications such as hematoma, dissection and perforation (12, 13). Perforation is rare but severe, leading to radial, cervical, mammary or mediastinal hematoma (9, 14–16). Nonetheless most doctors are reluctant to report their complications. Artery perforation occurs in <1.0% of patients undergoing transradial coronary interventions (9, 17, 18). Immediate recognition of the complications and prompt corrective actions are of utmost importance to prevent fatal outcomes. Prevention is better than cure for minimizing injury (19). A combination of changing directions and avoiding unwilling passages is a promising method to improve the safety and efficiency. We investigated a novel method, applying an active knuckle-angle 0.035-inch hydrophilic guidewire (knuckle guidewire) to prevent guidewire unwilling passage and related complications.

## Materials and Methods

### Study Population

Patients who underwent a transradial coronary procedure (both elective and emergent) in our team from August 2015 to June 2020 were retrospectively investigated. Among the included patients, 103 underwent emergent coronary angiography. Demographic and interventional characteristics of 1,457 patients receiving advancement of unmodified guidewires (Traditional group) and 1,322 patients receiving advancement of the knuckle guidewire (Knuckle group) were compared to investigate the safety of knuckle guidewire advancement. Afterwards, 239 patients were randomized according to a random number table to either the unmodified or the knuckle guidewire to further evaluate the efficiency of this novel method. The study protocol was approved by the ethics committee on Nov.12, 2018 (No. B2018-263) in Zhongshan Hospital, Fudan University.

### Transradial Puncture

All procedures were performed by two experienced operators via the radial arteries. The selected arm was positioned appropriately and sterilized. After local subcutaneous anesthesia with 1% lidocaine, radial artery puncture and a 6F hydrophilic sheath (Radiofocus Introducer II, Terumo, Tokyo, Japan) were introduced. After sheath insertion, 100 microgram (mcg) nitroglycerin was given to prevent vasospasm, and subsequently, 2,500 IU heparin diluted in 10 ml saline was injected into the radial artery.

### Active Knuckle-Angle 0.035-Inch Hydrophilic Guidewire (Knuckle Guidewire)

**Figure 1:**
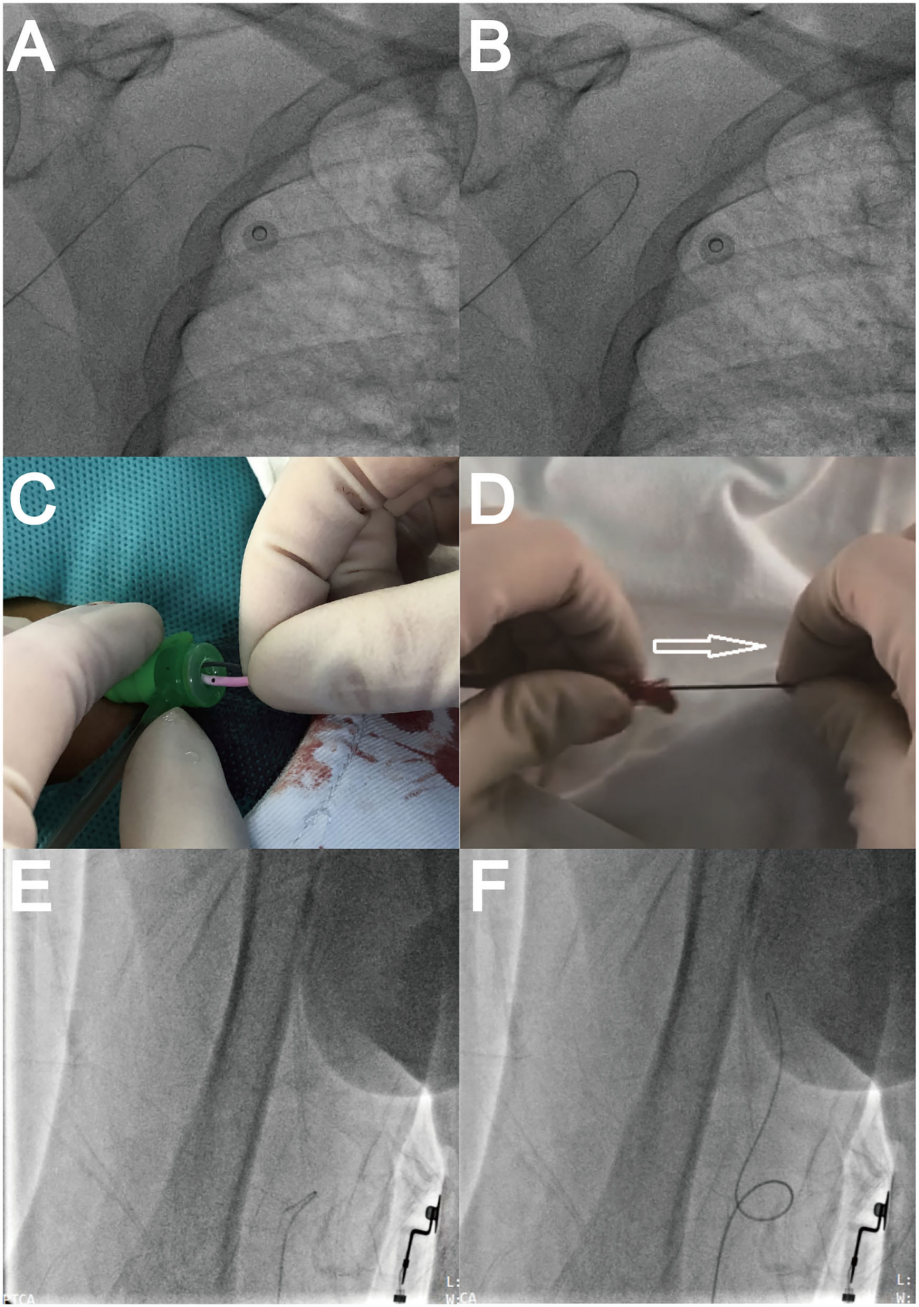
Guidewire performances. **(A)** Guidewire advancement. **(B)** Guidewire slipping into a branch artery. **(C)** Inserting 5F Tiger catheter and protruding guidewire for knuckle use. **(D)** Fixing guidewire tail and pulling back the catheter to loosen guidewire. **(E)** Knuckle guidewire advancement. **(F)** Passing artery loop.

Guidewire and catheter advancement were monitored with fluoroscopy at all times. The angle guidewire had a higher tendency to enter small side branches, even in experienced operators, and sometimes it was difficult to direct the guidewire back into the main branch (Supplementary Video 1). The occasional formation of knuckle guidewires, similar to J tips, avoided inadvertent slipping into branches. This inspired us to improve the guidewire advancement techniques (Figures 1A,B).

A knuckle guidewire operation was developed and is illustrated in Figure 1. First, the guidewire was advanced until a 1.5~2.0 cm protrusion was observed outside the 5F Tiger catheters (Terumo, Tokyo, Japan). Then, the catheter tip and protruding guidewire were manually inserted together into but not beyond the sheath (Figure 1C). Next, the guidewire tail was fixed, and the catheter was pulled back 2~3 cm to loosen the guidewire from the catheter in the sheath (Figure 1D). Finally, the knuckle guidewire was advanced to the aortic root under fluoroscopy (Figures 1E,F, Supplementary Video 2).

A 5F Tiger catheter was the first choice in coronary angiography (CAG), as it was difficult to insert a 6F catheter into the 6F sheath with a knuckle guidewire. Judkins or Amplatz catheters were used in the cases of unsuccessful selective cannulation of the coronary arteries with Tiger catheters.

The frequency of the guidewire entering side branches was recorded. Hemorrhage, arteriovenous fistula, and pseudoaneurysm related to the puncture site were not recorded, as they were associated with the puncture and hemostasis process. Perforation was defined as the rupture of the artery wall characterized by contrast extravasation demonstrated by angiography during the transradial procedure. All patients were followed up during their hospital stay. The primary outcome was the safety of the knuckle guidewire relative to the traditional guidewire (including guidewire unwilling passage and perforation). The secondary outcome was the efficiency of the knuckle guidewire (duration of time).

### Statistical Analysis

Variables are presented as the mean ± SD or as absolute numbers (percentage). Data analysis was carried out using SPSS version 22.0 (IBM, Armonk, New York). A 2-tailed *p* < 0.05 was considered to indicate statistical significance. Fisher's exact test was used for categorical variables. Student's *t-*test was used to compare continuous variables.

## Results

### Baseline Characteristics

Table 1 shows the comparison of the demographic characteristics of 1,457 patients receiving advancement of unmodified guidewires (Traditional group) and 1,322 patients receiving advancement of the knuckle guidewire (Knuckle group) in the retrospective investigation. Patients in the Knuckle group were more often male and had higher prevalence of hyperlipidemia, diabetes mellitus and smoking. No significant differences were identified between the two groups in other clinical characteristics, such as age, history of coronary artery disease and treatment, and left ventricular systolic function.

**Table 1:** Demographic characteristics.

	**Knuckle group** ***n* = 1,322**	**Traditional group** ***n* = 1,457**	***P*-value**
**Male (%)**	**881 (66.6)**	**901 (61.8)**	**0.008**
Age (years old)	63 ± 10	63 ± 9	0.51
Hypertension (%)	723 (54.7)	783 (53.7)	0.62
**Hyperlipidemia (%)**	**133 (10.1)**	**90 (6.2)**	**<0.001**
**Diabetes mellitus (%)**	**306 (23.1)**	**275 (18.9)**	**0.006**
**Smoking (%)**	**464 (35.1)**	**438 (30.1)**	**0.005**
Prior myocardial infarction (%)	122 (9.2)	136 (9.3)	0.92
Family history (%)	27 (2.0)	35 (2.4)	0.52
Chronic kidney dysfunction (%)	27 (2.0)	18 (1.2)	0.09
Prior percutaneous coronary intervention (%)	263 (19.9)	275 (18.9)	0.50
Prior coronary artery bypass graft (%)	8 (0.6)	19 (1.3)	0.15
Left ventricular ejection fraction (%)	62.3 ± 8.8	62.3 ± 8.4	0.99

*Items in bold indicate statistically significant differences*.

### Interventional Characteristics

Table 2 illustrates the procedural features in the Knuckle and Traditional group in the retrospective analysis. The puncture sites were similar between the groups. Small knuckle guidewires inadvertently slipping into the right common carotid artery occurred in only 1 patient in the Knuckle group. Unwilling passage of guidewires into side branches occurred in 139 (9.5%) patients in the Traditional group due to its inherent slippery nature, including once in 76 patients, twice in 28 patients, three times in 27 patients, four times in 5 patients, and up to five times in 3 patients.

**Table 2:** Interventional characteristics.

	**Knuckle group** ***n* = 1,322**	**Traditional group** ***n* = 1,457**	***P*-value**
Transradial access	1,350	1,468	0.38
Right radial artery (%)	1,264 (93.6)	1,386 (94.4)	
Left radial artery (%)	86 (6.4)	82 (5.6)	
**Guidewire unwilling passage (%)**	**1 (0.08)**	**139 (9.5%)**	**<0.001**
Once (%)	1 (100.0)	76 (54.7)	
Twice (%)	0	28 (20.1)	
Three times (%)	0	27 (19.4)	
Four times (%)	0	5 (3.6)	
Five times and more (%)	0	3 (2.2)	
**Identified vascular loops (%)**	**13 (1.0)**	**5 (0.3)**	**<0.001**
**Involved branches**			
Radial recurrent artery (%)	0 (0.0)	5 (0.3)	0.03
Internal mammary artery (%)	0 (0.0)	10 (0.7)	0.003
Common carotid artery (%)	1 (0.08)	13 (0.9)	0.002
**Perforation (%)**	**0 (0.0)**	**2 (0.1)**	**0.18**
Covered stent	0	1	
Compression	0	1	

*Items in bold indicate statistically significant differences*.

The radial recurrent artery always originates from the radioulnar alpha loop and runs parallel to the radial artery, which is the first branch to be inadvertently strayed into and this is easily missed during empirical advancement of the guidewire under fluoroscopy. Five patients complained of discomfort when the catheter passed the elbow via the guidewire in the Traditional group. Consequent angiography confirmed that the radial artery originated from a radioulnar loop and ran a similar parallel course to the recurrent radial artery, and the guidewire mistakenly entered the radial recurrent artery. We finally passed and straightened the loop successfully by adjusting the guidewire and catheter to perform CAG. The radioulnar loop was easily recognized and crossed in the Knuckle group. No knuckle guidewire slipped into the radial recurrent artery due to its small caliber and relatively large diameter of the knuckle guidewire. The internal mammarian artery (IMA) runs close to the sternum and parallel to the ascending aorta, making it another branch easily entered by error. In addition, atherosclerotic narrowing and tortuosity at the innominate-subclavian artery junction increases the risk of inadvertent insertion and requires a meticulous manipulation of the guidewire. In the Traditional group, guidewires inadvertently slipped into the IMA in 10 patients and into the common carotid artery in 13 patients.

**Figure 2:**
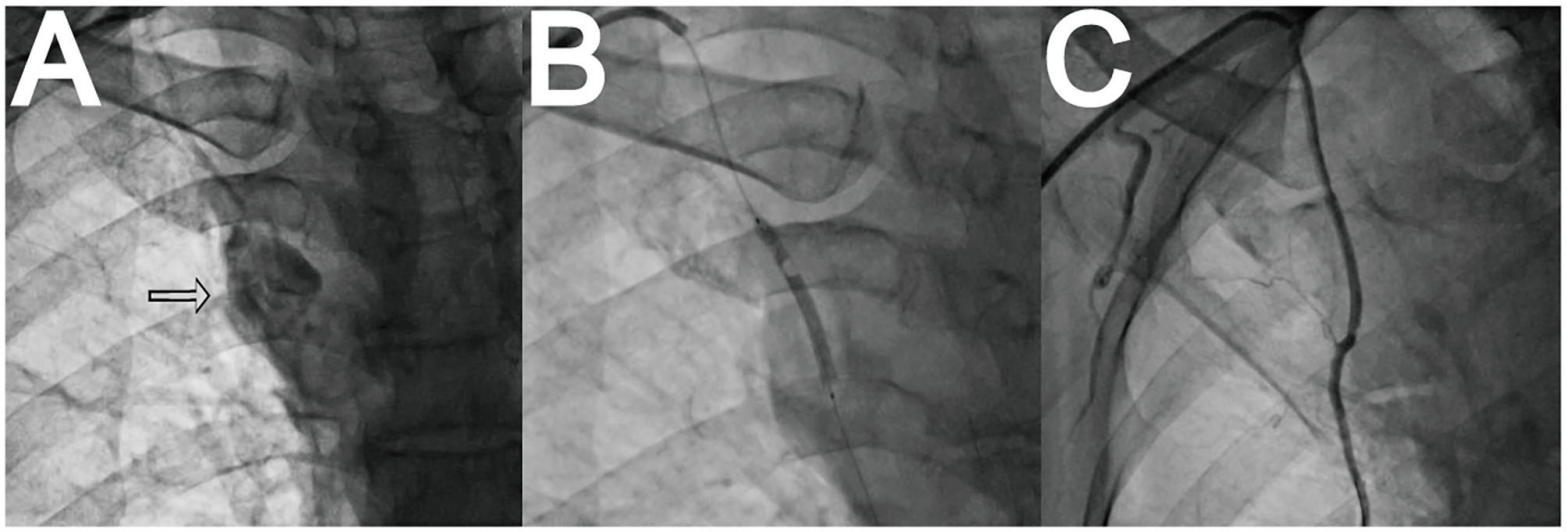
Unexpected perforation of right internal mammarian artery (RIMA) and treatment. **(A)** RIMA perforation. **(B)** Covered stent implantation. **(C)** Perforation sealed.

IMA perforation occurred in 1 patient and was treated with a covered stent (Figure 2, Supplementary Videos 3–5). No curved vessels or loops were observed.

Another patient suffered from brachial branch perforation with upper arm swelling and was treated by compression. She was a 67-year-old woman with a curved vessel but no loop.

In the Knuckle group the guidewire entered into the right common carotid artery in only 1 patient due to its small knuckle, causing no complications.

All patients were discharged from the hospital successfully.

### Efficiency Investigation

Considering the improved safety and fewer unwilling passages of knuckle guidewires, we designed a randomized controlled study to investigate its efficiency afterwards. 239 patients were randomly allocated (according to a random number table) to advance a guidewire with the knuckle method (*n* = 119) or the traditional method (*n* = 120). Matched baseline characteristics are presented in Table 3, including sex, age, concomitant diseases, and a history of coronary artery disease. Arterial loops between the two groups showed no differences and they were all crossed and straightened successfully. Primary and secondary endpoints are presented in Table 4. Unwilling passage of guidewires occurred more frequently in the Traditional group, and consequently, the need to re-adjust the guidewire increased the fluoroscopy time and dose. We calculated the median fluoroscopy time of guidewire advancement from sheath to aortic root and identified a significant decrease in the Knuckle group of 21 (ranging from 5 to 50) s relative to the Traditional group of 33 (ranging from 15 to 120) s. No guidewire-related complications occurred in either group, and the patients were discharged from the hospital successfully.

**Table 3:** Baseline characteristics of safety and efficiency investigation.

	**Knuckle group** ***n* = 119**	**Traditional group** ***n* = 120**
Male (%)	69 (58.0)	81 (67.5)
Age (years old)	62 ± 11	61 ± 10
Hypertension (%)	71 (59.7)	63 (52.5)
Hyperlipidemia (%)	15 (12.6)	14 (11.7)
Diabetes mellitus (%)	25 (21.0)	25 (20.8)
Smoking (%)	45 (37.8)	37 (30.8)
Prior myocardial infarction (%)	16 (13.4)	8 (6.7)
Prior percutaneous coronary intervention (%)	17 (14.3)	18 (15.0)
Prior coronary artery bypass graft (%)	1 (0.8)	2 (1.7)
Left ventricular ejection fraction (%)	64.1 ± 7.0	62.4 ± 8.2
**Transradial access**		
First time puncture (%)	92 (77.3)	96 (80.0)
Right radial artery (%)	115 (96.6)	112 (93.3)
Loops (%)	2 (1.7)	2 (1.7)

**Table 4:** Endpoints of safety and efficiency investigation.

	**Knuckle group** ***n* = 119**	**Traditional group** ***n* = 120**	***P*-value**
**Guidewire unwilling passage (%)**	**0 (0.0)**	**13 (10.8)**	**<0.001**
Perforation (%)	0 (0.0)	0 (0.0)	NA
Durations (s)^*^	**21 (5–50)**	**33 (15–120)**	**<0.001**

* *Duration of time is presented as “median (range)”. Items in bold indicate statistically significant differences*.

## Discussion

The incidence of unwilling passage of a guidewire is as high as 9.5% in transradial CAG, and its related perforations are rare but serious. In this study, we reported a novel method of advancing the hydrophilic guidewire with a modifiable tip: (1) advancement of the guidewire until a 1.5~2.0 cm protrusion outside the 5F Tiger catheters; (2) the catheter tip and protruding guidewire manually inserted together into but not beyond the sheath; (3) fix the guidewire tail and pull the catheter back 2~3 cm to loosen the guidewire from the catheter in the sheath; (4) advance the knuckle guidewire to the aortic root under fluoroscopy. In our analysis, unwilling passage of guidewires seldom occurred in the Knuckle group, thus improving the safety. A shorter fluoroscopy duration in the Knuckle group also contributed to a reduction in radiation exposure and greater efficiency.

A high incidence of unwilling passage of guidewires has not been reported for various reasons, but it is an inevitable problem in daily practice. Based on a similar parallel course of the pericardiacophrenic artery (7) and radial recurrent artery (20) to the normal arteries, the guidewire can inadvertently enter by mistake at some point during repeated manipulation without early recognition of error. Sometimes hydrophilic guidewires may slip into the same branch recurrently and it can be difficult to adjust them to follow the correct direction.

Meticulous advancement and unrecognized abnormalities could result in dissection and perforation. Early recognition and prompt action may prevent fatal outcomes. Asymptomatic dissection might be easily ignored without routine angiography and engage in self-healing over time (21). Immediate recognition of a perforation and prompt action including neutralization with heparin, crossing with a wire, and deployment of either a diagnostic or guide catheter across and external compression by a sphygmomanometer cuff, may help seal the perforation. Furthermore, invasive solutions, including prolonged balloon inflation, embolization, and covered stents, should be applied according to the patient's hemostatic response when the non-invasive treatments have obviously failed (7, 9, 17, 18). Although the incidence of perforation is low (9, 22), its consequences may be serious and even fatal for the patient (7, 13). And prevention is always more effective than a cure.

We designed a novel approach to prevent guidewire-associated unwilling passage and complications. Knuckle guidewires exhibit some superiorities, such as a preference for the main artery, not small branches and smooth tips friendly to the vessel wall (23, 24). A predefined knuckle could avoid slipping into the small branches, and the knuckle may enlarge and be unfastened once within a large luminal caliber. The recurrent radial artery branch always originates from the radioulnar alpha and runs parallel to the radial artery, making it easy to stray into without noticing while under fluoroscopy. Maneuvering during catheter passage may cause pain, vessel spasm, and even perforation (6, 9, 17). Most loops could be easily crossed and straightened using knuckle guidewire, while it is sometimes difficult to cross the loop by the traditional method due to multiple branching patterns along the loop (11). The IMA is far smaller than and nearly perpendicular to the subclavian artery, which prompts the knuckle guidewire to remain away from the IMA.

Frequently, male sex and a higher prevalence of hyperlipidemia, diabetes mellitus and smoking predicted more atherosclerosis in the Knuckle group, which could cause difficult passage of J-tip guidewires (15). However, the knuckle guidewire exhibited equal passage with the original angle hydrophilic guidewire. Smooth tips could be more friendly to the vessel wall than the original angle tips, especially when encountering tortuosity and loops. An angle tip contacting with the vessel wall increases the risk of dissection and perforation (9, 10, 17). Thanks to its preference for the main artery and a smooth tip, advancement of a knuckle guidewire becomes more efficient. Fluoroscopy dose is linearly correlated with fluoroscopy time, so a shorter duration of guidewire advancement will protect both the doctor and patient due to decreased radiation exposure.

Some interventional doctors may prefer the J-tip guidewire, whose tip is somewhat similar to the knuckle guidewire, with decreased entry into branches. However, it was originally designed for a large lumen in the transfemoral approach and has revealed some limitations in transradial access. First, the safe J tip only forms when it enters a large vessel or encounters a branch. However, before that, the tip remains straight in the small radial artery, so the potential risk of unwilling passage remains (17). Furthermore, the J tip is smaller than our knuckle tip, so it could also enter branches and lead to perforations (6, 12, 17, 25). Finally, it is sometimes difficult to pass tortuosity and severe angles of the subclavian-innominate-aorta axis using J-tip guidewires, which was dealt with by creating the hydrophilic angle guidewire (7, 15). Based on our experience, the knuckle guidewire could enlarge and unfasten its knuckle once in the large lumen, allowing it to possess the ability of directional changes to accomplish angiography in patients (Supplementary Video 6). Where there is a wire, there may be a complication! We should still pay more attention to its manipulation. Recent study proposed a risk core system (MATRIX score) to predict the radial crossover among patients with acute coronary syndrome managed invasively. This tool may provide guidance in anticipating potential difficulties related to transradial procedures and improve outcomes (26), which could be tried in future clinical practice.

### Study Limitations

There are some limitations of this study. This is a single-center and retrospective study. The enrolled patients were generally young with limited comorbidities, leading to possible selection bias. However, in this study, no perforation occurred in 605 patients over 70 years old, while 2 patients suffered severe complications in the traditional group, both of whom were 67 years old. This indicated that age might not be a risk factor for complications. The incidence of guidewire-related perforation was low, and the sample size was relatively small. Although a significant difference was not reached, we thought that the knuckle technique without unwilling passage of guidewires into branches could decrease the possibility of guidewire-associated complications. In addition, a knuckle guidewire with changes in its original tip may influence its use.

## Conclusion

Unwilling passage of guidewire occurred in 9.5% of cases in transradial angiography, and consequent perforation was rare but serious. Active knuckle-angle guidewires represented a novel method to prevent unwilling passage and associated perforation, contributing to greater efficiency and a reduction in radiation exposure.

## Data Availability Statement

The raw data supporting the conclusions of this article will be made available by the authors, without undue reservation.

## Ethics Statement

The studies involving human participants were reviewed and approved by the Ethics Committee on Nov.12, 2018 (No. B2018-263) in Zhongshan Hospital, Fudan University. The patients/participants provided their written informed consent to participate in this study.

## Author Contributions

HY, JC, and FZ conceived the idea and design for the study. MZ, CL, and YD analyzed the data. HY, LX, and JC drafted the manuscript. JQ provided guidance and suggestions throughout the entire study. All authors contributed to interpret the data, revise the draft critically for important intellectual content, and approved the final manuscript.

## Funding

This work was supported by National Natural Science Foundation of China (81801374 HY, 82070320 JC, and 82100466 LX), Shanghai Clinical Research Center for Interventional Medicine (19MC1910300), Shanghai Rising Stars of Medical Talent Youth Development Program (Shanghai Municipal Health Commission 2019-72 HY), and Shanghai Sailing Program (18YF1404800 LX).

## Conflict of Interest

The authors declare that the research was conducted in the absence of any commercial or financial relationships that could be construed as a potential conflict of interest.

## Publisher's Note

All claims expressed in this article are solely those of the authors and do not necessarily represent those of their affiliated organizations, or those of the publisher, the editors and the reviewers. Any product that may be evaluated in this article, or claim that may be made by its manufacturer, is not guaranteed or endorsed by the publisher.

## Supplementary Material

Supplementary Video 1 Guidewire slipped into branch arteries recurrently.

Supplementary Video 2 Knuckle guidewire passed and straightened artery loop easily.

Supplementary Video 3 Perforation of right internal mammarian artery.

Supplementary Video 4 Covered stent implantation.

Supplementary Video 5 Complete sealing of the perforation.

Supplementary Video 6 Knuckle guidewire unfastened its knuckle spontaneously and passed the tortuosity at innominate-subclavian artery junction.

## Abbreviations

CAG: coronary angiography: knuckle guidewire, Active knuckle angle 0.035-inch hydrophilic guidewire
IMA: internal mammarian artery.

## References

[1] Andò G Capodanno D. Radial access reduces mortality in patients with acute coronary syndromes: results from an updated trial sequential analysis of randomized trials. JACC Cardiovasc Interv. (2016) 9:660–70. 10.1016/j.jcin.2015.12.008 27056303

[2] Jolly SS Yusuf S Cairns J Niemelä K Xavier D Widimsky P. Radial versus femoral access for coronary angiography and intervention in patients with acute coronary syndromes (RIVAL): a randomised, parallel group, multicentre trial. Lancet. (2011) 377:1409–20. 10.1016/S0140-6736(11)60404-2 21470671

[3] Valgimigli M Gagnor A Calabró P Frigoli E Leonardi S Zaro T. Radial versus femoral access in patients with acute coronary syndromes undergoing invasive management: a randomised multicentre trial. Lancet. (2015) 385:2465–76. 10.1016/S0140-6736(15)60292-6 25791214

[4] Romagnoli E Biondi-Zoccai G Sciahbasi A Politi L Rigattieri S Pendenza G. Radial versus femoral randomized investigation in ST-segment elevation acute coronary syndrome: the RIFLE-STEACS (radial versus femoral randomized investigation in ST-elevation acute coronary syndrome) study. J Am Coll Cardiol. (2012) 60:2481–9. 10.1016/j.jacc.2012.06.017 22858390

[5] Bernat I Horak D Stasek J Mates M Pesek J Ostadal P. ST-segment elevation myocardial infarction treated by radial or femoral approach in a multicenter randomized clinical trial: the STEMI-RADIAL trial. J Am Coll Cardiol. (2014) 63:964–72. 10.1016/j.jacc.2013.08.1651 24211309

[6] Buturak A Demirci Y Dagdelen S. Management of an iatrogenic radial artery perforation: a case report. Turk Kardiyol Dern Ars. (2013) 41:332–5. 10.5543/tkda.2013.56957 23760121

[7] Arsanjani R Echeverri J Movahed MR. Successful coil embolization of pericardiacophrenic artery perforation occurring during transradial cardiac catheterization via right radial artery. J Invasive Cardiol. (2012) 24:671–4. 23220984

[8] Barbeau GR. Radial loop and extreme vessel tortuosity in the transradial approach: advantage of hydrophilic-coated guidewires and catheters. Catheter Cardiovasc Interv. (2003) 59:442–50. 10.1002/ccd.10586 12891603

[9] Tatli E Buturak A Cakar A Vatan BM Degirmencioglu A Agac TM. Unusual vascular complications associated with transradial coronary procedures among 10,324 patients: case based experience and treatment options. J Interv Cardiol. (2015) 28:305–12. 10.1111/joic.12206 25989895

[10] Sharma A Rajvanshi S Kumar T Pandit N. Large pectoral haematoma post-transradial catheterisation: an unusual but avoidable complication. BMJ Case Rep. (2017) 8:bcr2017221088. 10.1136/bcr-2017-221088 28835427 PMC5624020

[11] Farman MT Khan NU Rizvi SN. Successful transradial percutaneous coronary intervention with radial artery anomaly. J Pak Med Assoc. (2010) 60:593–5. 20578618

[12] Sanmartín M Cuevas D Goicolea J Ruiz-Salmerón R Gómez M Argibay V. Vascular complications associated with radial artery access for cardiac catheterization. Rev Esp Cardiol. (2004) 57:581–4. 10.1016/S0300-8932(04)77150-X 15225506

[13] Guo J Song J. A hydrophilic-wire induced vascular perforation causing mediastinal hematoma during transradial coronary intervention. J Invasive Cardiol. (2019) 31:E96. 31034444 10.25270/jic/18.00283

[14] Park KW Chung JW Chang SA Kim KI Chung WY Chae IH. Two cases of mediastinal hematoma after cardiac catheterization: a rare but real complication of the transradial approach. Int J Cardiol. (2008) 130:e89–92. 10.1016/j.ijcard.2007.05.093 17673316

[15] Ghori MA Al Zubaidi A Khwaja A. Thyrocervical trunk perforation: a rare vascular complication during cardiac intervention through right radial approach: a case report and literature review. J Saudi Heart Assoc. (2019) 31:121–4. 10.1016/j.jsha.2019.03.002 31031551 PMC6479075

[16] Jao YT Chen Y Fang CC Wang SP. Mediastinal and neck hematoma after cardiac catheterization. Catheter Cardiovasc Interv. (2003) 58:467–72. 10.1002/ccd.10476 12652496

[17] Sallam MM Ali M Al-Sekaiti R. Management of radial artery perforation complicating coronary intervention: a stepwise approach. J Interv Cardiol. (2011) 24:401–6. 10.1111/j.1540-8183.2011.00649.x 21539609

[18] Al-Sekaiti R Ali M Sallam M. Radial artery perforation after coronary intervention: is there a role for covered coronary stent? Catheter Cardiovasc Interv. (2011) 78:632–5. 10.1002/ccd.22945 21780276

[19] Mamas MA Fraser DG Ratib K Fath-Ordoubadi F El-Omar M Nolan J. Minimising radial injury: prevention is better than cure. EuroIntervention. (2014) 10:824–32. 10.4244/EIJV10I7A142 24472679

[20] Yoon SE Park S Ahn SG. Successful transradial intervention via a radial recurrent artery branch from the radioulnar alpha loop using a sheathless guiding catheter. Yeungnam Univ J Med. (2018) 35:94–8. 10.12701/yujm.2018.35.1.94 31620577 PMC6784667

[21] Valsecchi O Vassileva A Musumeci G Rossini R Tespili M Guagliumi G. Failure of transradial approach during coronary interventions: anatomic considerations. Catheter Cardiovasc Interv. (2006) 67:870–8. 10.1002/ccd.20732 16649233

[22] Tizón-Marcos H Barbeau GR. Incidence of compartment syndrome of the arm in a large series of transradial approach for coronary procedures. J Interv Cardiol. (2008) 21:380–4. 10.1111/j.1540-8183.2008.00361.x 18537873

[23] Ogura T Yamada M Nishioka N Yamada T Higuchi K. Gastrointestinal: Knuckle guidewire insertion: safe techniques of guidewire insertion into the pancreatobiliary tract using a novel 0.025-inch guidewire. J Gastroenterol Hepatol. (2020) 35:707. 10.1111/jgh.14895 31828835

[24] Ogura T Yamada M Yamada T Ueno S Higuchi K. Reverse knuckle guidewire insertion technique for endoscopic ultrasound-guided hepaticogastrostomy using a novel 0.025-inch guidewire. Endoscopy. (2020) 52:E418–9. 10.1055/a-1149-8738 32330952

[25] Goel S Cordeiro N Friedman M. Radial artery perforation complicating percutaneous coronary intervention. Cardiovasc Revasc Med. (2019) 20:26–7. 10.1016/j.carrev.2019.03.013 30971333

[26] Gragnano F Jolly SS Mehta SR Branca M van Klaveren D Frigoli E. Prediction of radial crossover in acute coronary syndromes: derivation and validation of the MATRIX score. Eurointervention. (2021) 17:971–80. 10.4244/EIJ-D-21-00441 34374343 PMC9724886

